# Secretion of Milk Suspended by Sassafras Tea

**Published:** 1836

**Authors:** N. Field

**Affiliations:** Jeffersonville, Indiana


### Art. III.—The Secretion of Milk suspended by Sassafras Tea.

*Experiments on the power of the Laurus Sassafras in suspending the secretion of Milk in the human mammæ*, by Dr. N. Field, of Jeffersonville, Indiana.

During the autumn of 1830, in consequence of wounding my hand in the dissection of a man who died of pneumonia, a singular eruption appeared on the affected hand and arm, which gradually extended itself over my whole body. For this strange and anomalous disease, I used liberally the blue pill, sarsaparilla and sassafras tea. My wife was at the time suckling our second child, and frequently participated with me in the use of the sassafras tea, which was regularly served up at breakfast and supper, and for which she had a considerable fondness. After the lapse of two or three days, she observed a diminution in the quantity of her milk, accompanied by an unusual flaccidity of the breasts. Not knowing to what cause the reduction of milk was attributed, she still continued to drink the sassafras tea until the secretion was totally suspended. The inconvenience to the child at that time, only three months old, as well as the mother, induced me to investigate the cause of so novel an occurrence with more care than I otherwise should have done. The thought struck my mind that it might possibly be occasioned by the sassafras tea. I accordingly advised my wife to discontinue its use, which she did. In two days the milk began to return, and by the fourth or fifth day after she ceased to drink it, the breast contained the customary quantity. To be certain that it was the effect of the sassafras, I got her to resume the use of it. In two or three days the secretion was greatly diminished with the like flaccidity of the mammæ. The tea was again laid aside, upon which the milk was again secreted. This experiment was repeated three or four times with precisely the same results. I have since recommended its use in cases where the breasts were likely to become inflamed from a redundancy of milk which so often occurs immediately after parturition, and, indeed, in many cases where the breasts were so inflamed and distended as to render the abstraction of the milk, by artificial means, extremely painful. I have found the most decided benefit to result from its use; and, in every case, a rapid diminution of the secretion was the consequence.

My observations on this subject induce me to believe, that the best mode of preparation is a strong infusion of the bark of the root.

*Jeffersonville, Indiana, September*, 1836.

